# Metabolic Traits and Risk of Ischemic Stroke in Japanese and European Populations: A Two-Sample Mendelian Randomization Study

**DOI:** 10.3390/metabo14050255

**Published:** 2024-04-27

**Authors:** Jinxia Zhang, Huimin Lu, Mingyang Cao, Jie Zhang, Di Liu, Xiaoni Meng, Deqiang Zheng, Lijuan Wu, Xiangdong Liu, Youxin Wang

**Affiliations:** 1 School of Public Health, Capital Medical University, Beijing 100069, China; jinxiaz@our.ecu.edu.au (J.Z.); luhuimin@mail.ccmu.edu.cn (H.L.); caomingyang97@163.com (M.C.); zhangjie@ccmu.edu.cn (J.Z.); meng123@mail.ccmu.edu.cn (X.M.); dqzheng@ccmu.edu.cn (D.Z.); xiaowu@ccmu.edu.cn (L.W.); 2 Beijing Key Laboratory of Clinical Epidemiology, Capital Medical University, Beijing 100069, China; 3 Centre for Biomedical Information Technology, Shenzhen Institutes of Advanced Technology, Chinese Academy of Sciences, Shenzhen 518055, China; 4 National Institute for Viral Disease Control and Prevention, Chinese Center for Disease Control and Prevention, Beijing 100069, China; 5 School of Public Health, North China University of Science and Technology, Tangshan 063210, China

**Keywords:** mendelian randomization, metabolic traits, ischemic stroke, different races, causal inference

## Abstract

The role of metabolic traits in ischemic stroke (IS) has been explored through observational studies and a few Mendelian randomization (MR) studies employing limited methods in European populations. This study aimed to investigate the causal effects of metabolic traits on IS in both East Asian and European populations utilizing multiple MR methods based on genetic insights. Two-sample and multivariable MR were performed, and MR estimates were calculated as inverse-variance weighted (IVW), weighted median, and penalized weighted median. Pleiotropy was assessed by MR–Egger and Mendelian randomization pleiotropy residual sum and outlier tests. Systolic blood pressure (SBP) was associated with an increased risk of IS by IVW in both European (OR*_IVW_*: 1.032, 95% CI: 1.026–1.038, *p* < 0.001) and Japanese populations (OR*_IVW_*: 1.870, 95% CI: 1.122–3.116, *p* = 0.016), which was further confirmed by other methods. Unlike the European population, the evidence for the association of diastolic blood pressure (DBP) with IS in the Japanese population was not stable. No evidence supported an association between the other traits and IS (all *P*s > 0.05) in both races. A positive association was found between SBP and IS in two races, while the results of DBP were only robust in Europeans.

## 1. Introduction

The burden of mortality as well as disability caused by stroke increases over time. Data from the Global Burden of Disease Study indicate that, in all age groups, stroke ranked as the fifth leading cause of disability-adjusted life years (DALYs) in 1990, ascending to the third position by 2019 [1]. Among all types of strokes, ischemic stroke (IS) accounts for the highest proportion [2]. Given that the majority of IS incidents are preventable [3], elucidating its etiological factors is of paramount importance. Observational studies have found that metabolic traits such as blood pressure, blood lipids, and blood glucose are risk factors for stroke. Moreover, these metabolic traits are mostly components of metabolic syndrome (MetS), a cluster of abnormal metabolic conditions regarded as an important risk factor for IS. Due to the diversity of its components, which include central obesity, dyslipidemia, increased blood pressure, and high blood glucose (BG) levels, the association of MetS and the metabolic traits with IS has gradually become a concern [4,5,6,7,8]. However, the limitations of traditional epidemiology approaches, combined with the substantial costs associated with conducting randomized controlled trials and comorbidities among metabolic traits and other risk factors, significantly increase the difficulty of inferring a causal relationship between metabolic traits and IS [9].

Mendelian randomization (MR) is increasingly utilized to investigate causality between modifiable risk factors and cardiovascular diseases [10], especially when confounding is a significant concern. MR leverages genetic variants (such as single-nucleotide polymorphisms (SNPs)) as instrumental variables (IVs) to estimate the effect of exposure on an outcome. Given that genetic variants are randomly allocated at birth, bias caused by reverse causation or other potential confounders can be circumvented [11]. Pleiotropy, the phenomenon wherein genetic variants affect the outcome via multiple independent biological pathways, is one of the potential factors that can contribute to bias in MR studies [12]. To guard against the bias caused by unknown shared factors, Morrison J. et al. proposed a new MR method termed causal analysis using summary effect estimates (CAUSE), which can distinguish causality from correlated pleiotropy [13].

Several MR studies have investigated the causal associations between specific metabolic traits and stroke risk. Notably, a two-sample MR study elucidated the significant effects of metabolic disorders, comprising abdominal obesity, insulin resistance, and so forth, on the risk of stroke [14]. Another MR demonstrated that both SBP and DBP were causally associated with the risk of IS [15]. Furthermore, the results of several studies regarding blood lipids in this regard exhibit heterogeneity [16,17]. However, the majority of the existing relevant MR studies were carried out in Europeans and mainly focused on central obesity-related indicators [18,19,20]. Although it is advocated to apply multiple MR methods in two-sample MR studies [21], previous studies mainly focused on the results from inverse-variance weighted (IVW), weighted median (WM), penalized weighted median (PWM), and MR–Egger methods [15,16,17,22,23]. Based on this, our study aimed to use summary-level data from the Japanese population to elucidate the association between metabolic traits and IS by employing both two-sample and multivariable MR. Additionally, to mitigate bias arising from pleiotropy, our findings were verified by various MR methods. Beyond that, we extended our analysis to the European population employing the CAUSE method to enhance the robustness of the causality evidence.

## 2. Materials and Methods

### 2.1. Data Source

#### 2.1.1. Japanese Population

The outcome data of the Japanese population utilized in this study were sourced from the genome-wide association study (GWAS) provided by the Japanese ENcyclopedia of GEnetic associations by the Riken (JENGER) site. This GWAS encompassed 42 diseases based on 212,453 Japanese individuals and detected 383 independent signals in 331 loci for 30 diseases [24]. The database of IS included 17,671 case samples as well as 192,383 control samples, of which the cases were collected in the BioBank Japan Project (BBJ) and the control samples were from population-based prospective cohorts. The case group consisted of 11,081 males with an average age of 69.8 years. The control group included 97,455 male participants with a mean age of 61.3 years, and those with cerebral aneurysms were excluded. Ethical approval for the study was granted by the relevant ethics committee, and informed consent was obtained from all participants.

Exposure data were also extracted from GWAS on the JENGER site. This study identified 1407 trait–associated loci for 53 quantitative traits in 162,255 Japanese individuals, of which 679 loci were discovered for the first time [25]. A few MR studies have explored the relationship between obesity-related indicators and IS; the GWAS referenced in our research did not include summary-level data pertaining to waist circumference. Consequently, based on available data, we included BG (*n* = 93,146), systolic blood pressure (SBP, *n* = 136,597), diastolic blood pressure (DBP, *n* = 136,615), triglycerides (TG, *n* = 105,597), total cholesterol (TC, *n* = 128,305), low-density lipoprotein cholesterol (LDL–C, *n* = 72,866), and high-density lipoprotein cholesterol (HDL–C, *n* = 70,657) as exposures. Written, informed consent was obtained from all participants, and the study was approved by the relevant ethics committees.

#### 2.1.2. European Population

Summary data for IS were obtained from the Multiancestry genome-wide association study (MEGASTROKE), a fixed-effects meta-analysis restricted to Europeans (including 40,585 cases and 406,111 controls) [26]. A total of 446,696 individuals, including 34,217 cases of IS, were included in the pooled data of IS in the European population only.

Data on BP, BG, and blood lipids were obtained from the Genome-Wide Repository of Associations between SNPs and Phenotypes (GRASP, n = 757,601) [27], the Meta-Analyses of Glucose and Insulin-related traits Consortium (MAGIC, n = 151,188) [28], and Global Lipids Genetics Consortium (GLGC, n = 188,578) [29], respectively. GRASP pooled data from the UK Biobank (UKB) and the International Consortium of Blood Pressure Genome-Wide Association Studies (ICBP). After excluding subjects with significant missing data, a total of 757,601 participants were finally included in the study. MEGASTROKE was conducted in a European population after excluding individuals diagnosed with diabetes who were receiving treatment for diabetes and whose fasting plasma glucose was greater than or equal to 7 mmol/L. The genetic effects on glucose and insulin were analyzed under sex-dimorphic and sex-combined conditions. In this study, we utilized sex-specific pooled GWAS data, which included 151,188 individuals. GLGC investigated the genetic determinants of LDL–C, HDL–C, and TG in the blood to understand the genetic causes associated with lipid quantitative traits. Participants in the pooled data were also drawn entirely from the European population, which included 188,578 participants post-exclusion of individuals who were taking lipid-lowering drugs. Ultimately, 157 lipid-level–related sites were identified that met the genome-wide significance threshold (*p* < 5 × 10^−8^), of which 62 were found for the first time. The TC, TG, LDL–C, and HDL–C data used in this study were derived exclusively from this study.

### 2.2. SNP Selection

All SNPs associated with BG, SBP, DBP, TC, TG, LDL–C, and HDL–C at the genome-wide significance level (*p* < 5 × 10^−8^) were selected. By using the 1000 Genomes East Asian or European ancestry reference panel, linkage disequilibrium (LD) was clumped (distance threshold = 10 000 kb; r^2^ = 0.001) to ensure the independence of SNPs. Table S1 shows the SNP information after filtering. However, to mitigate potential confounding due to the correlation among selected exposures, SNPs that overlapped across traits were systematically excluded. To assess the effect of overlapping SNPs, MR analysis was performed on the data prior to exclusion. In addition, the *R*^2^ of each SNP was calculated based on the values provided and formula $R^{2}=2\times MAF\times (1-MAF)\times {Beta}^{2}$; the F-statistic of each exposure was then obtained according to the formula $F=\frac{R^{2}}{1-R^{2}}\times \frac{N-K-1}{K}$, where *K* represents the number of IVs. The bias caused by weak IVs was controlled according to the rule of thumb (*F* < 10 is regarded as a weak instrumental variable). The characteristics of the SNPs post-exclusion are summarized in Table S2.

### 2.3. MR Analyses

MR was based on the following three assumptions: (1) Genetic variants are associated with exposure, which ensures that genetic variants can effectively proxy for exposure. A strong genetic association increases the instrument’s validity and the precision of causal estimates. (2) Genetic variants are not associated with confounding factors that bias the associations between exposure and outcome. It is essential to avoid confounding in MR studies as any link between the genetic variant and other factors that affect both the exposure and the outcome could bias the results. We ensured this by selecting variants identified through rigorous GWAS that are specific to the exposure. (3) Genetic variants influence outcomes only via their association with exposure. The exclusivity of this path is crucial for direct causal inference. If it is violated, it could suggest alternative pathways affecting the outcome, potentially confounding the causal estimate. We assessed this through sensitivity analyses to ensure the integrity of our causal interpretation. The IVW method, commonly regarded as the conventional MR method, rigorously adheres to these assumptions and gives accurate estimates when genetic variants have no pleiotropic effects [30]. Heterogeneity among SNPs was estimated by the Cochran Q statistic. Random effects IVW models were employed if heterogeneity existed, whereas a fixed effects IVW model was utilized otherwise. To mitigate potential pleiotropy, we also performed complementary MR analyses using WM, PWM, MR–Egger, Mendelian randomization pleiotropy residual sum and outlier (MR–PRESSO), and the CAUSE method. MR–Egger can be used to detect and adjust the bias caused by directional pleiotropy under a weaker set of assumptions but is less efficient than IVW and WM [31]. WM ensures consistency when 50% of the weight contributed by genetic variants is valid, while PWM behaves akin to weighted median when there is no causal effect heterogeneity [32]. To minimize false positives as much as possible, two MR methods were further performed for the exposure that was statistically significant in the IVW results. MR–PRESSO was additionally utilized to identify and rectify horizontal pleiotropy through the removal of outliers [33]. Furthermore, CAUSE incorporates information from all variants, instead of only those most strongly associated with exposure to differentiate causal effects from correlated pleiotropy, thus avoiding more false positives [13]. Low power caused by insufficient inclusion of IVs is a pervasive issue encountered in MR. To avoid false-negative results related to insufficient power, the MR–robust adjusted profile score (MR–RAPS) method, which can increase the statistical power when some IVs exhibit substantial strength while many remain weak, was deployed for exposures with *p* values greater than 0.05 [34]. Furthermore, three thresholds of 5 × 10^−6^, 1 × 10^−6^, and 5 × 10^−8^ were set for selecting exposure-related SNPs in MR–RAPS analyses to ensure the reliability of negative results. In consideration of the potential association among metabolic traits, we included multiple exposures through multivariable Mendelian randomization (MVMR) to discern their independent effects on IS [35].

**Figure 1:**
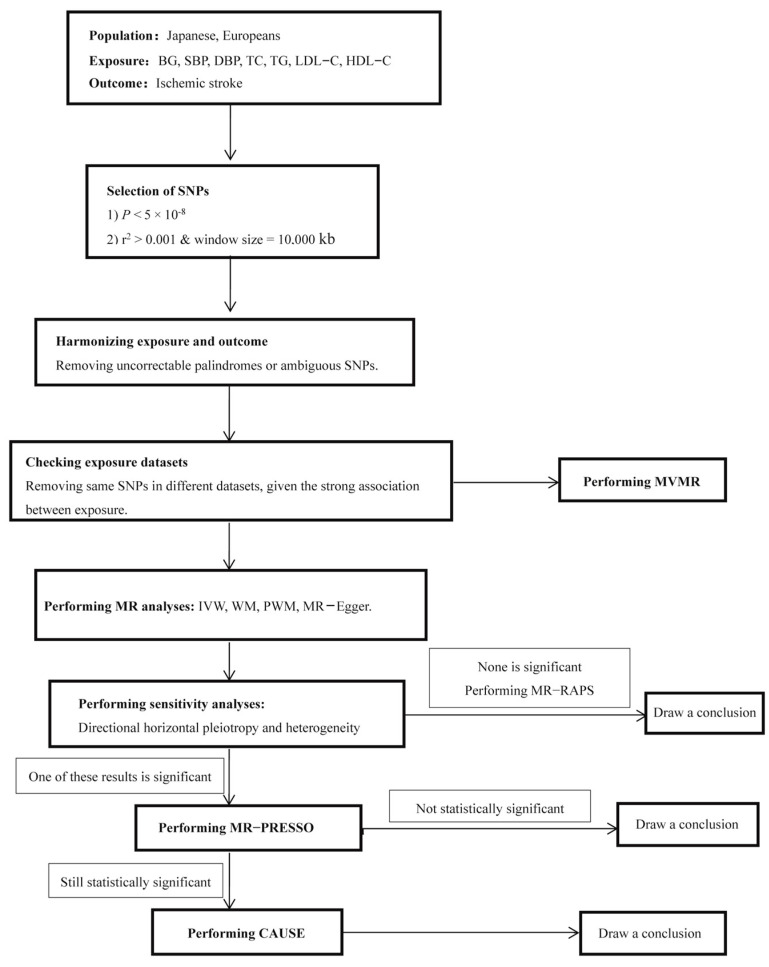
Flow chart for genetic variant selection and two-sample MR analyses. MR, Mendelian randomization; BG, blood glucose; SBP, systolic blood pressure; DBP, diastolic blood pressure; TC, total cholesterol; TG, triglyceride; LDL-C, low-density lipoprotein cholesterol; HDL-C, high-density lipoprotein cholesterol; IS, ischemic stroke; SNP, single-nucleotide polymorphism; IVW, inverse-variance weighted method; WM, weighted median method; PWM, penalized weighted median method; MR–Egger, Egger regression method; MR-PRESSO, Mendelian randomization pleiotropy residual sum and outlier test; CAUSE, causal analysis using summary effect estimates; MR-RAPS, MR-robust adjusted profile score.

In terms of sensitivity analyses, MR–Egger was used to assess directional pleiotropy. Under a power of 80%, the minimally (for Beta > 0) or maximally (for Beta < 0) detectable effect size for MR of each exposure with IS was estimated by an online platform (https://shiny.cnsgenomics.com/mRnd/, accessed on 15th February 2022) [36]. For positive results, the type Ⅰ error rate was calculated through an online platform (https://sb452.shinyapps.io/overlap, accessed on 15th February 2022) to evaluate the possible bias caused by sample overlap [37]. A flow chart for genetic variant selection and two-sample MR analyses is shown in Figure 1.

Statistical tests were all two–tailed. For the CAUSE and MVMR methods, *p* < 0.05 was used as the significance threshold, while for the other methods, the significance threshold (*p* < 0.05/7 = 0.007) after Bonferroni correction was considered statistically significant, and results with *p* values between 0.007 and 0.05 were considered suggestive evidence. The analyses were performed in R version 4.1.0.

## 3. Results

### 3.1. Results Description

#### 3.1.1. Japanese Population

Following the exclusion of overlapping SNPs, 11 BG–associated SNPs, 12 SBP–associated SNPs, 7 DBP–associated SNPs, 30 TC–associated SNPs, 18 TG–associated SNPs, 19 LDL–C–associated SNPs, and 37 HDL–C–associated SNPs were included in MR analyses, and the *F* statistic of each component was greater than 30, indicating that the results were not affected by the bias of weak IVs. Under 80% power, the detectable effect size for each exposure is presented in Table S3.

As the conventional MR method, IVW was first applied to our analyses. Among the results of IVW, evidence for causal association was not found in the analyses of BG (odds ratio (OR): 1.036, 95% confidence interval (CI): 0.848–1.265, *p* = 0.731), DBP (OR: 1.966, 95% CI: 0.829–4.663, *p* = 0.125), TC (OR: 1.044, 95% CI: 0.914–1.193, *p* = 0.523), TG (OR: 0.992, 95% CI: 0.915–1.074, *p* = 0.840), LDL–C (OR: 1.038, 95% CI: 0.907–1.189, *p* = 0.585), or HDL–C (OR: 0.961, 95% CI: 0.893–1.034, *p* = 0.285); only the *p* value of SBP (OR: 1.870, 95% CI: 1.122–3.116, *p* = 0.016) was less than 0.05 but still did not meet the significance threshold after Bonferroni corrections (Table 1). However, prior to the exclusion of overlapping SNPs, both IVW and PWM found a statistical association of SBP and DBP with IS (Table S4). For BG, TG, TC, HDL, and LDL, after raising the inclusion threshold and including more SNPs, all the *p* values in the MR–RAPS analyses were still greater than 0.05, suggesting that the results of these traits were unlikely to be false negatives caused by insufficient power (Table S5).

Taking into account the heterogeneity, other MR methods were then performed. In the PWM analyses, not only SBP was shown to be associated with a higher risk for IS (OR: 2.446, 95% CI: 1.575–3.799, *p* < 0.001) but DBP was also identified as a contributing factor to the risk of IS (OR: 3.597, 95% CI: 1.832–7.062, *p* < 0.001), while the *p* values of other traits were still greater than 0.05 (Table 2).

After the exclusion of outliers, the MR–PRESSO results no longer supported the causal association between DBP and IS (*p* = 0.230). Nevertheless, the association between SBP and IS was still robust (OR: 2.168, 95% CI: 1.470–3.198, *p* = 0.004). Additionally, the distortion test showed that there was no significant difference in causal estimates before and after outlier correction (Table 3).

In the CAUSE analysis, the causal model of both SBP and DBP demonstrated superior performance compared to the shared model, but only the *p* value of SBP was less than 0.05 (*p* = 0.042, Table 4). MVMR methods, after controlling the exposures with overlapping SNPs, further substantiated the association between SBP and IS (all *P*s < 0.05), while evidence for DBP was observed only in the MVMR–median method (*p* = 0.041, Table S6). Nonetheless, when lipid exposures were included in the model, no evidence for an association with IS was observed across both MVMR methods (Table S7).

In the sensitivity analyses (Table S3), horizontal pleiotropy was not detected in any analyses between each exposure and IS (all *P*s > 0.05). However, heterogeneity was found in SBP, DBP, and LDL–C (*p* < 0.05). Consequently, a random effect model was adopted in IVW, and PWM results rather than WM results were considered.

#### 3.1.2. European Population

Before eliminating overlapping SNPs, analyses in the European population revealed that the *p* values of SBP (OR: 1.036, 95% CI: 1.030–1.041), DBP (OR: 1.052, 95% CI: 1.043–1.061), TC (OR: 1.118, 95% CI: 1.032–1.211), and LDL–C (OR: 1.101, 95% CI: 1.026–1.181) with IS were all less than 0.05 by IVW, which was also consistent with the results obtained from other methods (Table S8).

A total of 38 SNPs for BG, 324 SNPs for SBP, 319 SNPs for DBP, 34 SNPs for TC, 45 SNPs for TG, 33 SNPs for LDL–C, and 68 SNPs for HDL–C were left after SNP control. Despite the reduction in IVs included, in the results of the IVW method as well as the other three methods, SBP (OR*_IVW_*= 1.032, 95% CI: 1.026–1.038) and DBP (OR*_IVW_*= 1.044, 95% CI: 1.033–1.054) remained statistically significant with IS (Table 5). Furthermore, IVW only identified evidence of an association with HDL–C; however, the association was not further verified by other methods, while the association with SBP and DBP was further demonstrated in the other three MR methods, including WM, PWM, and MR–Egger.

Given the limited genetic variations in BG and TG indexes in the European population and that the results are always negative, we opted to relax the inclusion criteria for genetic variations and use the MR–RAPS method to determine whether the negative results were attributable to insufficient test efficiency. Upon adjusting the inclusion threshold to encompass more SNPs, no association between BG or TG and IS was observed by the MR–RAPS method (*p* > 0.05, Table S9).

In consideration of heterogeneity, other MR methods were then performed. In the PWM analyses, both SBP (OR: 1.033, 95% CI: 1.025–1.041, *p* < 0.001) and DBP (OR: 1.046, 95% CI: 1.032–1.061, *p* < 0.001) were shown to be significantly associated with a higher risk for IS. Furthermore, TC was also identified to play a role in the risk of IS (OR: 1.220, 95% CI: 1.017–1.463, *p* = 0.032), while the *p* values of other traits were still greater than 0.05 (Table 6).

After correcting for outliers, MR–PRESSO results showed that SBP, DBP, and HDL–C were still statistically significant in relation to IS (*p* < 0.05). The distortion test revealed no significant difference between the results before and after outlier correction (*p* > 0.05, Table 7).

In the CAUSE analysis, the causal model of both SBP and DBP was superior to the shared model, and the difference was statistically significant (*p* < 0.05, Table S10). For the MVMR methods after controlling the exposures with overlapping SNPs, consistent with the results from the MVMR analysis in the Japanese population, the results in the European population further corroborated the association between SBP and IS (all *P*s < 0.05) while evidence for DBP was observed only in the MVMR–Median method (*p* = 0.021, Table S11). However, when lipid exposures were included in the model, no evidence supporting an association with IS was found for either MVMR method (Table S12). In the sensitivity analysis (Table S13), heterogeneity was still observed in SBP, DBP, TC, LDL–C, and HDL–C (*p* < 0.05), but PWM did not lend support to causality.

## 4. Discussion

This study explored the causal relationships of seven metabolic traits with IS based on Japanese and European populations using multiple MR methods. SBP was identified to be causally associated with an increased risk of IS, and a similar result was observed in DBP analyses of Europeans, while it was not robust in Japanese analyses. In addition, no evidence was found to support the causal role of other metabolic traits in IS.

BP plays an important role in vascular function and organ perfusion and is also the most common clinical symptom recorded at stroke presentation [38]. Observational studies have underscored the critical importance of BP in the pathogenesis, development, and prognosis of stroke [39,40,41]. In a two-sample MR analyses carried out in a European population (over 400 SNPs included), both SBP and DBP were identified as causal factors in the risk of IS [15]. A bidirectional MR study in a European population showed that hypertension was associated with an increased risk of IS [8]. Further, another MR study on a European population, incorporating in excess of 300 SNPs for analyses, further corroborated its causal association [22]. Even after exclusion, the association was still observed in the PWM results. Beyond that, results from randomized controlled trials (RCTs) showed that BP lowering can reduce the risk of stroke [42,43]. Similar results were also observed in our study. For Europeans, both before and after SNP control, SBP and DBP were statistically associated with IS; for Japanese individuals, the association between SBP and IS risk was found to be robust, while DBP was only supported by WM and PWM methods, and the results were no longer positive when pleiotropy was further controlled by the CAUSE method, which seemed inconsistent with the results from RCTs. This inconsistency results between MR and RCTs also appeared in other previous studies [44,45]. In addition to the multitarget effect of the intervention in RCTs, pleiotropy may account for the difference between RCTs and MR, which poses a threat to the robustness as well as reliability of MR [46]. It is noteworthy that the SNPs included in our study were far outnumbered by those in European MR analyses, which led to a higher proportion of variance in BP being explained by the IV in European compared to ours, and this may account for the lack of observed association between BP and IS that was not observed in our MVMR analyses. Although the CAUSE method further controls the issue of false positive, the power of CAUSE is lower than that of other MR methods (especially IVW and WM) in many settings presented in its simulation analyses [13]. Due to the limited number of SNPs and the issue of CAUSE, our results need to be further verified by a larger East Asian sample as well as CAUSE results based on Europeans.

Traditional epidemiological studies have underscored the association between lipids and the risk of IS [47,48,49], and this correlation is also supported by some robust evidence in MR studies [50]. Although one European-based MR study found evidence for LDL–C, instead of TG or HDL–C, with an increased risk of IS [16], another MR study only found suggestive evidence (results with *p* value below 0.05 but above Bonferroni-corrected *p*) for LDL–C and TC with IS after further controlling for LD [17]. The existence of LD will violate the third assumption of MR and lead to false-positive results, which may account for the variations observed in the above studies. For our study, r^2^ < 0.001 was also used for LD, and different thresholds for IV selection were applied considering insufficient power. In the European population examined in this study, certain specific MR methods suggested a relationship between LDL–C and TC with IS. However, other MR methods have shown heterogeneity in the results, which may be attributed to the different algorithms employed by these MR methods. Furthermore, no causal association was observed in the MVMR analysis, possibly due to the presence of horizontal pleiotropy for LDL–C and heterogeneity for both. Furthermore, it is noteworthy that previous studies [16,17] incorporated IS subtypes for analyses and found that the association of lipids with large artery stroke is more obvious than that with all IS. A study of an African population provides evidence of a causal effect of lipid traits on the risk of IS in individuals of African descent [7]. Considering the differences among stroke subtypes, further studies covering more IS types should be carried out in East Asians.

Contrary to a previous MR study [23], no association between BG and IS risk was found in this study. Although previous research suggested that glucose control is widely regarded as beneficial in IS prevention [51,52], RCTs found that intensive therapy targeting normal glycated hemoglobin levels did not significantly reduce cardiovascular events but increased mortality [53,54,55]. Combined with current MR studies, the effect of glucose control in IS prevention may stem more from the effect of the treatment regimen on other body indicators rather than a causal relationship between the two. Moreover, further study on the association between HbA1c levels and IS in East Asians is imperative to better explore the difference in the role of HbA1c in different races and understand the relationship between T2D and IS.

### Strengths and Limitations

This study is the first to explore the causal association between the seven metabolic traits and IS in a large East Asian population from genetic insights; various MR methods were utilized to control for possible bias caused by pleiotropy, and we included more comprehensive metabolic traits. The study acknowledges certain limitations, particularly concerning the limited number of available SNPs: (1) Given that participants of the exposed group also came from the BBJ database, a certain degree of overlap with the case participants in the outcome group is possible. However, the sample size of the control group is large enough, which makes the overlap ratio low. Moreover, given the negative MR results of this study, the potential type I error inflated by sample overlap does not change our conclusion. (2) The stringent criteria applied during SNP selection led to the inclusion of a limited number of SNPs, which resulted in a low proportion of each component’s variation explained by SNPs, representing a significant challenge in MR research. (3) Pleiotropy is another challenge encountered in MR analyses. Currently, MR methods for detecting and correcting pleiotropy are limited, and new methods are required to further validate our results. (4) This study relied on summary-level data from public databases, so limited data are available to further analyze the association of metabolic traits with IS subtypes. Additionally, the results of this study also need to be further verified by data from other East Asian populations.

## 5. Conclusions

Based on large-scale Japanese and European summary data, this study explored the causal association between seven metabolic traits and IS by employing various two-sample MR methods from genetic insights. Our study found robust evidence for the association between SBP and IS in two races. However, contrasting with the results in Europeans, the evidence of DBP and IS in Japanese individuals was not robust enough. Moreover, no evidence was found to support the causal association of other traits and IS.

## Figures and Tables

**Table 1:** IVW results for seven metabolic traits on IS among the Japanese population.

Exposure	SNP (N)	OR (95% CI)	Beta (SE)	*p*
BG	11	1.036 (0.848–1.265)	0.035 (0.102)	0.731
SBP	12	1.870 (1.122–3.116)	0.626 (0.261)	0.016 *
DBP	7	1.966 (0.829–4.663)	0.676 (0.441)	0.125
TC	30	1.044 (0.914–1.193)	0.043 (0.068)	0.523
TG	18	0.992 (0.915–1.074)	0.008 (0.041)	0.840
LDL–C	18	1.038 (0.907–1.189)	0.038 (0.069)	0.585
HDL–C	37	0.961 (0.893–1.034)	0.040 (0.038)	0.285

Beta is the estimated effect size. IVW, inverse-variance weighted; IS, ischemic stroke; BG, blood glucose; SBP, systolic blood pressure; DBP, diastolic blood pressure; TC, total cholesterol; TG, triglyceride; LDL–C, low-density lipoprotein cholesterol; HDL–C, high-density lipoprotein cholesterol; SNP, single-nucleotide polymorphism; OR, odds ratio; CI, confidence interval; SE, standard error; * *p* < 0.05, the result is statistically significant.

**Table 2:** Other MR results for seven metabolic traits on IS among the Japanese population.

Exposure	Method	SNP (N)	OR (95% CI)	Beta (SE)	*p*
BG	WM	11	1.029 (0.815–1.300)	0.029 (0.117)	0.806
	PWM	11	1.026 (0.815–1.293)	0.026 (0.118)	0.825
	MR–Egger	11	0.667 (0.252–1.763)	−0.404 (0.496)	0.436
SBP	WM	12	1.871 (1.234–2.836)	0.626 (0.212)	3.187 × 10^−3^ *
	PWM	12	2.446 (1.575–3.799)	0.894 (0.225)	6.854 × 10^−5^ *
	MR–Egger	12	0.732 (0.050–10.661)	−0.311 (1.366)	0.824
DBP	WM	7	2.182 (1.139–4.178)	0.780 (0.332)	0.019 *
	PWM	7	3.597 (1.832–7.062)	1.280 (0.344)	2.000 × 10^−4^ *
	MR–Egger	7	12.610 (0.506–313.959)	2.534 (1.640)	0.183
TC	WM	30	1.006 (0.835–1.213)	0.006 (0.095)	0.947
	PWM	30	1.005 (0.832–1.214)	0.005 (0.096)	0.961
	MR–Egger	30	1.109 (0.763–1.611)	0.104 (0.191)	0.591
TG	WM	18	0.969 (0.878–1.070)	−0.031 (0.050)	0.534
	PWM	18	0.969 (0.877–1.071)	−0.031 (0.051)	0.542
	MR–Egger	18	0.934 (0.828–1.049)	−0.070 (0.060)	0.263
LDL−C	WM	18	1.019 (0.858–1.210)	0.0189 (0.088)	0.829
	PWM	18	1.019 (0.848–1.225)	0.0189 (0.094)	0.840
	MR–Egger	18	1.036 (0.730–1.471)	0.035 (0.179)	0.846
HDL−C	WM	37	0.973 (0.882–1.074)	−0.027 (0.050)	0.586
	PWM	37	0.976 (0.883–1.077)	−0.024 (0.051)	0.625
	MR–Egger	37	1.026 (0.895–1.175)	0.026 (0.069)	0.715

Beta is the estimated effect size. MR, Mendelian randomization; IS, ischemic stroke; BG, blood glucose; SBP, systolic blood pressure; DBP, diastolic blood pressure; TC, total cholesterol; TG, triglyceride; LDL–C, low-density lipoprotein cholesterol; HDL–C, high-density lipoprotein cholesterol; SNP, single-nucleotide polymorphism; OR, odds ratio; CI, confidence interval; SE, standard error; WM, weighted median method; PWM, penalized weighted median method; MR–Egger, Egger regression method; * *p* < 0.05, the result is statistically significant.

**Table 3:** MR–PRESSO results for BP components on IS among the Japanese population.

Exposure	MR–Analysis	SNP (N)	OR (95% CI)	Beta (SE)	*P^a^*	*P^b^*	*P^c^*
SBP	Outlier-corrected	10	2.168 (1.470–3.198)	0.774 (0.198)	<0.001 *	0.004 *	0.503
DBP	Outlier-corrected	4	1.963 (0.771–4.994)	0.674 (0.477)	<0.001 *	0.230	0.956

*P^a^* is the value of *p* for the global test performed by the MR–PRESSO method to detect potential horizontal pleiotropy. *P^b^* is the value of *p* for MR–PRESSO analysis after outlier correction. *P^c^* is the value of *p* for the distortion test performed by the MR–PRESSO to test the significant differences in causal estimates before and after outlier correction. * *p* < 0.05, the result is statistically significant. MR–PRESSO, Mendelian randomization pleiotropy residual sum and outlier; BP, blood pressure; IS, ischemic stroke; SBP, systolic blood pressure; DBP, diastolic blood pressure; LDL–C, low-density lipoprotein cholesterol; SNP, single-nucleotide polymorphism; OR, odds ratio; CI, confidence interval; SE, standard error.

**Table 4:** CAUSE results for BP on IS among the Japanese population.

Exposure	SNP (N)	Model 1	Model 2	∆ELPD	SE of ∆ELPD	Z	*p*
SBP	1076	Null	Sharing	−5.854	2.772	−2.111	0.017 *
	1076	Null	Causal	−8.805	4.311	−2.042	0.021 *
	1076	Sharing	Causal	−2.950	1.708	−1.726	0.042 *
DBP	977	Null	Sharing	−8.479	3.631	−2.335	0.001 *
	977	Null	Causal	−10.830	4.894	−2.213	0.013 *
	977	Sharing	Causal	−2.351	1.605	−1.465	0.071

N is the number of variants used for estimating CAUSE posteriors. ∆ELPD = ELPD*_C_* − ELPD*_S_*. When ∆ELPD is negative, model 2 is a better fit. CAUSE, causal analysis using summary effect; ELPD, expected log pointwise posterior density; SE, standard error; SBP, systolic blood pressure; DBP, diastolic blood pressure. * *p* < 0.05, the result is statistically significant.

**Table 5:** IVW results for seven metabolic traits on IS among the European population.

Exposure	SNP (N)	OR (95% CI)	Beta (SE)	*p*
BG	38	1.077 (0.957–1.213)	0.075 (0.060)	0.217
SBP	324	1.032 (1.026–1.038)	0.032 (0.003)	1.748 × 10^−27^ *
DBP	319	1.044 (1.033–1.054)	0.043 (0.005)	2.623 × 10^−17^ *
TC	34	1.045 (0.917–1.192)	0.045 (0.067)	0.506
TG	45	1.015 (0.943–1.093)	0.015 (0.038)	0.687
LDL–C	33	1.070 (0.937–1.222)	0.068 (0.069)	0.319
HDL–C	68	0.880 (0.798–0.971)	−0.127 (0.050)	0.011 *

Beta is the estimated effect size. IVW, inverse–variance weighted; IS, ischemic stroke; BG, blood glucose; SBP, systolic blood pressure; DBP, diastolic blood pressure; TC, total cholesterol; TG, triglyceride; LDL–C, low-density lipoprotein cholesterol; HDL–C, high-density lipoprotein cholesterol; SNP, single-nucleotide polymorphism; OR, odds ratio; CI, confidence interval; SE, standard error; * *p* < 0.05, the result is statistically significant.

**Table 6:** Other MR results for seven metabolic traits on IS among the European population.

Exposure	Method	SNP (N)	OR (95% CI)	Beta (SE)	*p*
BG	WM	38	0.966 (0.866–1.077)	−0.035 (0.055)	0.533
	PWM	38	0.966 (0.863–1.081)	−0.035 (0.058)	0.548
	MR–Egger	38	0.925 (0.801–1.068)	−0.078 (0.073)	0.295
SBP	WM	324	1.033 (1.025–1.040)	0.032 (0.004)	1.141 × 10^−17^ *
	PWM	324	1.033 (1.025–1.041)	0.033 (0.004)	9.354 × 10^−16^ *
	MR–Egger	324	1.043 (1.026–1.060)	0.042 (0.008)	8.903 × 10^−7^ *
DBP	WM	319	1.046 (1.033–1.060)	0.045 (0.007)	1.331 × 10^−11^ *
	PWM	319	1.046 (1.032–1.061)	0.045 (0.007)	8.600 × 10^−11^ *
	MR–Egger	319	1.062 (1.034–1.091)	0.060 (0.014)	1.837 × 10^−5^ *
TC	WM	34	1.215 (1.025–1.441)	0.195 (0.087)	0.025 *
	PWM	34	1.220 (1.017–1.463)	0.199 (0.093)	0.032 *
	MR–Egger	34	1.102 (0.799–1.520)	0.097 (0.164)	0.559
TG	WM	45	1.008 (0.914–1.111)	0.007 (0.050)	0.880
	PWM	45	1.009 (0.916–1.110)	0.009 (0.049)	0.861
	MR–Egger	45	0.951 (0.844–1.071)	−0.051 (0.061)	0.411
LDL−C	WM	33	0.980 (0.837–1.146)	−0.021 (0.080)	0.796
	PWM	33	0.980 (0.830–1.156)	−0.021 (0.084)	0.804
	MR–Egger	33	1.094 (0.822–1.456)	0.090 (0.146)	0.543
HDL−C	WM	68	0.893 (0.794–1.004)	−0.113 (0.060)	0.059
	PWM	68	0.890 (0.789–1.003)	−0.116 (0.061)	0.058
	MR–Egger	68	1.145 (0.916–1.430)	0.135 (0.113)	0.238

Beta is the estimated effect size. MR, Mendelian randomization; IS, ischemic stroke; BG, blood glucose; SBP, systolic blood pressure; DBP, diastolic blood pressure; TC, total cholesterol; TG, triglyceride; LDL–C, low-density lipoprotein cholesterol; HDL–C, high-density lipoprotein cholesterol; SNP, single-nucleotide polymorphism; OR, odds ratio; CI, confidence interval; SE, standard error; WM, weighted median method; PWM, penalized weighted median method; MR–Egger, Egger regression method; * *p* < 0.05, the result is statistically significant.

**Table 7:** MR–PRESSO results for BP components and HDL–C on IS among the European population.

Exposure	MR–Analysis	SNP (N)	OR (95% CI)	Beta (SE)	*P^a^*	*P^b^*	*P^c^*
SBP	Outlier-corrected	318	1.033 (1.026–1.039)	0.032 (0.003)	<0.001 *	2.558 × 10^−26^ *	0.977
DBP	Outlier-corrected	314	1.044 (1.034–1.054)	0.043 (0.005)	<0.001 *	2.167 × 10^−17^ *	0.986
HDL–C	Outlier-corrected	67	0.899 (0.833–0.971)	−0.106 (0.039)	0.001 *	0.008	0.517

*P^a^* is the value of *p* for the global test performed by the MR–PRESSO method to detect potential horizontal pleiotropy. *P^b^* is the value of *p* for MR–PRESSO analysis after outlier correction. *P^c^* is the value of *p* for the distortion test performed by the MR–PRESSO to test the significant differences in causal estimates before and after outlier correction. * *p* < 0.05, the result is statistically significant. MR–PRESSO, Mendelian randomization pleiotropy residual sum and outlier; BP, blood pressure; IS, ischemic stroke; SBP, systolic blood pressure; DBP, diastolic blood pressure; HDL–C, high-density lipoprotein cholesterol; SNP, single-nucleotide polymorphism; OR, odds ratio; CI, confidence interval; SE, standard error.

## Data Availability

The data presented in this study are available in the link in the article and Supplementary Materials. The publicly available summary data on metabolic traits and ischemic stroke (IS) were provided by the Japanese ENcyclopedia of GEnetic associations by the Riken (JENGER) site, the BioBank Japan Project (BBJ), the Multiancestry genome-wide association study (MEGASTROKE), the Genome-Wide Repository of Associations between SNPs and Phenotypes (GRASP), the Meta-Analyses of Glucose and Insulin-related traits Consortium (MAGIC), and the Global Lipids Genetics Consortium (GLGC), respectively.

## Supplementary Materials

The following supporting information can be downloaded at https://www.mdpi.com/article/10.3390/metabo14050255/s1: Table S1. The characteristics of SNPs used for MR analyses in Japanese (before exclusion); Table S2. The characteristics of SNPs used for MR analyses in Japanese (after exclusion); Table S3. Horizontal pleiotropy and heterogeneity tests of seven metabolic traits and IS in Japanese; Table S4. MR results for seven metabolic traits on IS in Japanese (before exclusion); Table S5. MR–RAPS results for seven metabolic traits on IS in Japanese (before exclusion); Table S6. Multivariable results for five metabolic traits on risk of IS; Table S7. Multivariable results for four metabolic traits on risk of IS; Table S8. MR results for seven metabolic traits on IS in Europeans; Table S9. MR–RAPS results for seven metabolic traits on IS in Europeans (before exclusion); Table S10. CAUSE results for metabolic traits on IS in Europeans; Table S11. Multivariable results for three metabolic traits on risk of IS; Table S12. Multivariable results for four metabolic traits on risk of IS; Table S13. Horizontal pleiotropy and heterogeneity tests of seven metabolic traits and IS in Europeans.

## References

[1] GBD 2019 (2020). Global burden of 369 diseases and injuries in 204 countries and territories, 1990–2019: A systematic analysis for the Global Burden of Disease Study 2019. Lancet.

[2] Feigin V.L., Nguyen G., Cercy K., Johnson C.O., Alam T., Parmar P.G., Abajobir A.A., Abate K.H., Abd-Allah F., Abejie A.N. (2018). Global, Regional, and Country-Specific Lifetime Risks of Stroke, 1990 and 2016. N. Engl. J. Med..

[3] Diener H.-C., Hankey G.J. (2020). Primary and Secondary Prevention of Ischemic Stroke and Cerebral Hemorrhage: JACC Focus Seminar. J. Am. Coll. Cardiol..

[4] DeBoer M.D., Filipp S.L., Sims M., Musani S.K., Gurka M.J. (2020). Risk of Ischemic Stroke Increases Over the Spectrum of Metabolic Syndrome Severity. Stroke.

[5] Horn J.W., Feng T., Mørkedal B., Strand L.B., Horn J., Mukamal K., Janszky I. (2021). Obesity and Risk for First Ischemic Stroke Depends on Metabolic Syndrome: The HUNT Study. Stroke.

[6] Decker J.J., Norby F.L., Rooney M.R., Soliman E.Z., Lutsey P.L., Pankow J.S., Alonso A., Chen L.Y. (2019). Metabolic Syndrome and Risk of Ischemic Stroke in Atrial Fibrillation: ARIC Study. Stroke.

[7] Fatumo S., Karhunen V., Chikowore T., Sounkou T., Udosen B., Ezenwa C., Nakabuye M., Soremekun O., Daghlas I., Ryan D.K. (2021). Metabolic Traits and Stroke Risk in Individuals of African Ancestry: Mendelian Randomization Analysis. Stroke.

[8] He Q., Wang W., Li H., Xiong Y., Tao C., Ma L., You C. (2023). Genetic insights into the risk of metabolic syndrome and its components on stroke and its subtypes: Bidirectional Mendelian randomization. J. Cereb. Blood Flow Metab..

[9] Bozkurt B., Aguilar D., Deswal A., Dunbar S.B., Francis G.S., Horwich T., Jessup M., Kosiborod M., Pritchett A.M., Ramasubbu K. (2016). Contributory Risk and Management of Comorbidities of Hypertension, Obesity, Diabetes Mellitus, Hyperlipidemia, and Metabolic Syndrome in Chronic Heart Failure: A Scientific Statement From the American Heart Association. Circulation.

[10] Boehm F.J., Zhou X. (2022). Statistical methods for Mendelian randomization in genome-wide association studies: A review. Comput. Struct. Biotechnol. J..

[11] Emdin C.A., Khera A.V., Kathiresan S. (2017). Mendelian Randomization. JAMA.

[12] Cinelli C., LaPierre N., Hill B.L., Sankararaman S., Eskin E. (2022). Robust Mendelian randomization in the presence of residual population stratification, batch effects and horizontal pleiotropy. Nat. Commun..

[13] Morrison J., Knoblauch N., Marcus J.H., Stephens M., He X. (2020). Mendelian randomization accounting for correlated and uncorrelated pleiotropic effects using genome-wide summary statistics. Nat. Genet..

[14] Wang Z., Chen J., Zhu L., Jiao S., Chen Y., Sun Y. (2023). Metabolic disorders and risk of cardiovascular diseases: A two-sample mendelian randomization study. BMC Cardiovasc. Disord.

[15] Georgakis M.K., Gill D., Webb A.J.S., Evangelou E., Elliott P., Sudlow C.L.M., Dehghan A., Malik R., Tzoulaki I., Dichgans M. (2020). Genetically determined blood pressure, antihypertensive drug classes, and risk of stroke subtypes. Neurology.

[16] Hindy G., Engström G., Larsson S.C., Traylor M., Markus H.S., Melander O., Orho-Melander M. (2018). Role of Blood Lipids in the Development of Ischemic Stroke and its Subtypes: A Mendelian Randomization Study. Stroke.

[17] Yuan S., Tang B., Zheng J., Larsson S.C. (2020). Circulating Lipoprotein Lipids, Apolipoproteins and Ischemic Stroke. Ann. Neurol..

[18] Kim M.S., Kim W.J., Khera A.V., Kim J.Y., Yon D.K., Lee S.W., Shin J.I., Won H.-H. (2021). Association between adiposity and cardiovascular outcomes: An umbrella review and meta-analysis of observational and Mendelian randomization studies. Eur. Heart J..

[19] Marini S., Merino J., Montgomery B.E., Malik R., Sudlow C.L., Dichgans M., Florez J.C., Rosand J., Gill D., Anderson C.D. (2020). Mendelian Randomization Study of Obesity and Cerebrovascular Disease. Ann. Neurol..

[20] Si S., Tewara M.A., Li Y., Li W., Chen X., Yuan T., Liu C., Li J., Wang B., Li H. (2020). Causal Pathways from Body Components and Regional Fat to Extensive Metabolic Phenotypes: A Mendelian Randomization Study. Obesity.

[21] Yu Y., Hou L., Shi X., Sun X., Liu X., Yu Y., Yuan Z., Li H., Xue F. (2022). Impact of nonrandom selection mechanisms on the causal effect estimation for two-sample Mendelian randomization methods. PLoS Genet..

[22] Wan E.Y.F., Fung W.T., Schooling C.M., Au Yeung S.L., Kwok M.K., Yu E.Y.T., Wang Y., Chan E.W.Y., Wong I.C.K., Lam C.L.K. (2021). Blood Pressure and Risk of Cardiovascular Disease in UK Biobank: A Mendelian Randomization Study. Hypertension.

[23] Georgakis M.K., Harshfield E.L., Malik R., Franceschini N., Langenberg C., Wareham N.J., Markus H.S., Dichgans M. (2021). Diabetes Mellitus, Glycemic Traits, and Cerebrovascular Disease: A Mendelian Randomization Study. Neurology.

[24] Ishigaki K., Akiyama M., Kanai M., Takahashi A., Kawakami E., Sugishita H., Sakaue S., Matoba N., Low S.-K., Okada Y. (2020). Large-scale genome-wide association study in a Japanese population identifies novel susceptibility loci across different diseases. Nat. Genet..

[25] Kanai M., Akiyama M., Takahashi A., Matoba N., Momozawa Y., Ikeda M., Iwata N., Ikegawa S., Hirata M., Matsuda K. (2018). Genetic analysis of quantitative traits in the Japanese population links cell types to complex human diseases. Nat. Genet..

[26] Malik R., Chauhan G., Traylor M., Sargurupremraj M., Okada Y., Mishra A., Rutten-Jacobs L., Giese A.-K., van der Laan S.W., Gretarsdottir S. (2018). Multiancestry genome-wide association study of 520,000 subjects identifies 32 loci associated with stroke and stroke subtypes. Nat. Genet..

[27] Evangelou E., Warren H.R., Mosen-Ansorena D., Mifsud B., Pazoki R., Gao H., Ntritsos G., Dimou N., Cabrera C.P., Karaman I. (2018). Genetic analysis of over 1 million people identifies 535 new loci associated with blood pressure traits. Nat. Genet..

[28] Lagou V., Mägi R., Hottenga J.-J., Grallert H., Perry J.R.B., Bouatia-Naji N., Marullo L., Rybin D., Jansen R., Min J.L. (2021). Sex-dimorphic genetic effects and novel loci for fasting glucose and insulin variability. Nat. Commun..

[29] Willer C.J., Schmidt E.M., Sengupta S., Peloso G.M., Gustafsson S., Kanoni S., Ganna A., Chen J., Buchkovich M.L., Mora S. (2013). Discovery and refinement of loci associated with lipid levels. Nat. Genet..

[30] Tin A., Köttgen A. (2021). Mendelian Randomization Analysis as a Tool to Gain Insights into Causes of Diseases: A Primer. J. Am. Soc. Nephrol. JASN.

[31] Brown B.C., Knowles D.A. (2021). Welch-weighted Egger regression reduces false positives due to correlated pleiotropy in Mendelian randomization. Am. J. Hum. Genet..

[32] Bowden J., Davey Smith G., Haycock P.C., Burgess S. (2016). Consistent Estimation in Mendelian Randomization with Some Invalid Instruments Using a Weighted Median Estimator. Genet. Epidemiol..

[33] Verbanck M., Chen C.-Y., Neale B., Do R. (2018). Detection of widespread horizontal pleiotropy in causal relationships inferred from Mendelian randomization between complex traits and diseases. Nat. Genet..

[34] Zhao Q., Chen Y., Wang J., Small D.S. (2019). Powerful three-sample genome-wide design and robust statistical inference in summary-data Mendelian randomization. Int. J. Epidemiol..

[35] Cai J., He L., Wang H., Rong X., Chen M., Shen Q., Li X., Li M., Peng Y. (2022). Genetic liability for prescription opioid use and risk of cardiovascular diseases: A multivariable Mendelian randomization study. Addiction.

[36] Brion M.-J.A., Shakhbazov K., Visscher P.M. (2013). Calculating statistical power in Mendelian randomization studies. Int. J. Epidemiol..

[37] Burgess S., Davies N.M., Thompson S.G. (2016). Bias due to participant overlap in two-sample Mendelian randomization. Genet. Epidemiol..

[38] Yang P., Song L., Zhang Y., Zhang X., Chen X., Li Y., Sun L., Wan Y., Billot L., Li Q. (2022). Intensive blood pressure control after endovascular thrombectomy for acute ischaemic stroke (ENCHANTED2/MT): A multicentre, open-label, blinded-endpoint, randomised controlled trial. Lancet.

[39] Lewington S., Clarke R., Qizilbash N., Peto R., Collins R. (2002). Age-specific relevance of usual blood pressure to vascular mortality: A meta-analysis of individual data for one million adults in 61 prospective studies. Lancet.

[40] Heshmatollah A., Ma Y., Fani L., Koudstaal P.J., Ikram M.A., Ikram M.K. (2022). Visit-to-visit blood pressure variability and the risk of stroke in the Netherlands: A population-based cohort study. PLoS Med..

[41] Flint A.C., Conell C., Ren X., Banki N.M., Chan S.L., Rao V.A., Melles R.B., Bhatt D.L. (2019). Effect of Systolic and Diastolic Blood Pressure on Cardiovascular Outcomes. N. Engl. J. Med..

[42] Psaty B.M., Lumley T., Furberg C.D., Schellenbaum G., Pahor M., Alderman M.H., Weiss N.S. (2003). Health outcomes associated with various antihypertensive therapies used as first-line agents: A network meta-analysis. JAMA.

[43] Ettehad D., Emdin C.A., Kiran A., Anderson S.G., Callender T., Emberson J., Chalmers J., Rodgers A., Rahimi K. (2016). Blood pressure lowering for prevention of cardiovascular disease and death: A systematic review and meta-analysis. Lancet.

[44] Wang L., Qiao Y., Zhang H., Zhang Y., Hua J., Jin S., Liu G. (2020). Circulating Vitamin D Levels and Alzheimer’s Disease: A Mendelian Randomization Study in the IGAP and UK Biobank. J. Alzheimer’s Dis. JAD.

[45] Thompson W.D., Tyrrell J., Borges M.-C., Beaumont R.N., Knight B.A., Wood A.R., Ring S.M., Hattersley A.T., Freathy R.M., Lawlor D.A. (2019). Association of maternal circulating 25(OH)D and calcium with birth weight: A mendelian randomisation analysis. PLoS Med..

[46] Hemani G., Bowden J., Davey Smith G. (2018). Evaluating the potential role of pleiotropy in Mendelian randomization studies. Hum. Mol. Genet..

[47] Holmes M.V., Millwood I.Y., Kartsonaki C., Hill M.R., Bennett D.A., Boxall R., Guo Y., Xu X., Bian Z., Hu R. (2018). Lipids, Lipoproteins, and Metabolites and Risk of Myocardial Infarction and Stroke. J. Am. Coll. Cardiol..

[48] Pikula A., Beiser A.S., Wang J., Himali J.J., Kelly-Hayes M., Kase C.S., Yang Q., Seshadri S., Wolf P.A. (2015). Lipid and lipoprotein measurements and the risk of ischemic vascular events: Framingham Study. Neurology.

[49] Li W., Huang Z., Fang W., Wang X., Cai Z., Chen G., Wu W., Chen Z., Wu S., Chen Y. (2022). Remnant Cholesterol Variability and Incident Ischemic Stroke in the General Population. Stroke.

[50] Sun L., Clarke R., Bennett D., Guo Y., Walters R.G., Hill M., Parish S., Millwood I.Y., Bian Z., Chen Y. (2019). Causal associations of blood lipids with risk of ischemic stroke and intracerebral hemorrhage in Chinese adults. Nat. Med..

[51] Boden-Albala B., Cammack S., Chong J., Wang C., Wright C., Rundek T., Elkind M.S.V., Paik M.C., Sacco R.L. (2008). Diabetes, fasting glucose levels, and risk of ischemic stroke and vascular events: Findings from the Northern Manhattan Study (NOMAS). Diabetes Care.

[52] Hayward R.A., Reaven P.D., Wiitala W.L., Bahn G.D., Reda D.J., Ge L., McCarren M., Duckworth W.C., Emanuele N.V. (2015). Follow-up of glycemic control and cardiovascular outcomes in type 2 diabetes. N. Engl. J. Med..

[53] Gerstein H.C., Miller M.E., Byington R.P., Goff D.C., Bigger J.T., Buse J.B., Cushman W.C., Genuth S., IsmaiL-Beigi F., Grimm R.H. (2008). Effects of intensive glucose lowering in type 2 diabetes. N. Engl. J. Med..

[54] Johnston K.C., Bruno A., Pauls Q., Hall C.E., Barrett K.M., Barsan W., Fansler A., Van de Bruinhorst K., Janis S., Durkalski-Mauldin V.L. (2019). Intensive vs Standard Treatment of Hyperglycemia and Functional Outcome in Patients With Acute Ischemic Stroke: The SHINE Randomized Clinical Trial. JAMA.

[55] Bains N.K., Huang W., French B.R., Siddiq F., Gomez C.R., Qureshi A.I. (2023). Hyperglycemic control in acute ischemic stroke patients undergoing endovascular treatment: Post hoc analysis of the Stroke Hyperglycemia Insulin Network Effort trial. J. Neurointerv. Surg..

